# Supplementary material for: Developing Consensus Standard Operating Procedures (SOPs) to Evaluate New Types of Insecticide-Treated Nets
Source: Insects. 2021 Dec 21;13(1):7. doi: 10.3390/insects13010007 (PMC8778287; doi:10.3390/insects13010007)
Supplement: Supplementary file 1 [file insects-13-00007-s001.zip › Additional File S1_Developing consensus SOPs for evaluating next-generation ITNs_Supplementary tables and figures_005.pdf]

Additional File S1: Developing consensus SOPs for evaluating new types of insecticide treated nets - supplementary tables and figures

Table S1. List of WHO prequalified pyrethroid + PBO nets and their product specifications [3].

Net name	AIs	PBO location	Insecticide content
DuraNet Plus	Alpha-cypermethrin<br>PBO	Sides & roof	6.0 g/kg (270 mg/m^2) alpha-cypermethrin<br>2.2 g/kg (99 mg/m^2) PBO
VEERALIN	Alpha-cypermethrin<br>PBO	Sides & roof	6.0 g/kg (216 mg/m^2) alpha-cypermethrin<br>2.2 g/kg (79 mg/m^2) PBO
PermaNet 3.0	Deltamethrin<br>PBO	Roof	Roof:<br>4 g/kg deltamethrin<br>25 g/kg PBO<br>Sides (deltamethrin only):<br>2.8 g/kg for 75 denier (115 mg/m^2 for strengthened border, and 85 mg/m^2 for remaining side panel)<br>2.1 g/kg for 100 denier
Tsara Boost	Deltamethrin<br>PBO	Sides & roof	12% (120 mg/m^2) deltamethrin<br>44% (440 mg/m^2) PBO
Tsara Plus	Deltamethrin<br>PBO	Roof	Roof:<br>3g/kg (120mg/m^2) deltamethrin<br>11g/kg (440 mg/m^2) PBO<br>Sides:<br>2.5g/kg (100mg/m^2) deltamethrin
Olyset Plus	Permethrin<br>PBO	Sides & roof	2% w/w permethrin (20g/kg, 800 mg/m^2)<br>1% w/w PBO (10 g/kg, 400 mg/m^2)

Table S2. List of WHO prequalified pyrethroid + PPF nets and its product specifications [3].

Net name	Insecticides	Insecticide content
Royal Guard	Alpha-cypermethrin<br>Pyriproxyfen	● 120 denier: 5.5 g/kg alpha-cypermethrin and 5.5 g/kg pyriproxyfen<br>● 150 denier: 5.0 g/kg alpha-cypermethrin and 5.0 g/kg pyriproxyfen

Table S3. List of WHO prequalified pyrethroid + CFP net and its product specifications [3].

Net name	Insecticides	Insecticide content
Interceptor G2 (IG2)	Chlorfenapyr<br>Alpha-cypermethrin	● 200 mg/m^2 chlorfenapyr<br>● 100 mg/m^2 alpha-cypermethrin

References

[3] WHO, "Prequalified Vector Control Products | WHO - Prequalification of Medical Products (IVDs, Medicines, Vaccines and Immunization Devices, Vector Control)," 2020.

https://extranet.who.int/pqweb/vector-control-products/prequalified-product-list (accessed Aug. 02, 2021).
