# Supplementary material for: Developing Consensus Standard Operating Procedures (SOPs) to Evaluate New Types of Insecticide-Treated Nets
Source: Insects. 2021 Dec 21;13(1):7. doi: 10.3390/insects13010007 (PMC8778287; doi:10.3390/insects13010007)
Supplement: Supplementary file 1 [file insects-13-00007-s001.zip › Additional File S2_PBO ITN Durability - SOP.pdf]

I2I-SOP-001: Methods for monitoring the durability of dual AI insecticide-treated nets containing a pyrethroid plus piperonyl butoxide (PBO)

1. Purpose 1
2. Background 2
3. Materials & Equipment 2
4. Procedure 3
   4.1. Test mosquitoes 3
   4.2. Collection and storage of test net samples (for bioefficacy testing) 3
   4.3. Control net samples 4
   4.4. Cone bioassay setup 6
   4.5. Cone bioassay procedure 6
   4.6. Measured outcomes 7
5. Data priority list 8
6. Deviations from standard protocol 9
7. Supplementary data 9
8. Glossary of terms 9
9. References 9

1. Purpose

This standard operating procedure (SOP) describes the methods to determine the bioefficacy of the pyrethroid and piperonyl butoxide (PBO) components of insecticide-treated nets (ITNs) used under operational conditions. The process used to determine the methodology detailed in this SOP, and justifications for key methodological parameters can be found in 'I2I-MD-001: Durability monitoring method development: Dual AI insecticide-treated nets containing a pyrethroid plus piperonyl butoxide (PBO)'.

2. Background

Several ITNs containing pyrethroid plus PBO (pyrethroid + PBO nets) are prequalified (PQ) by the WHO (WHO, 2020). These nets products have different specifications. They contain different pyrethroid insecticides at various concentrations, and PBO is located on varying parts of the net (e.g. roof only). Monitoring the bioefficacy of the active ingredients (AI) in the nets is a vital part of establishing the durability of these nets under operational conditions.

3. Materials & Equipment

General

- Data collection sheets
- Lab coat
- Gloves
- Test pyrethroid + PBO nets
- Control untreated net
- Control new pyrethroid + PPO net
- Control new pyrethroid only net
- Aspirator (manual/electronic), separate for each insecticide
- Mosquito strains
- Pen/permanent markers

Collection and storage of net samples

- Net frame
- Scissors
- Paper labels
- Aluminium foil

Cone bioassay

- Tape
- Mosquito holding containers (e.g. paper cups covered with untreated netting held by elastic bands)
- Cone holding frame (x 2), with holes to hold standard WHO plastic cones
- Cone holder frame stand, which holds frame at 45°
- WHO plastic cones
- Binder clips or clamps

- Cotton wool or rubber stoppers
- Temperature and humidity data logger
- Timer
- 10% sucrose solution (e.g. sugar or honey and water)
- Cotton wool

4. Procedure

4.1. Test mosquitoes

- Use 2-to-5-day-old non-blood fed female *Anopheles* mosquitoes. Mosquitoes should be well characterized lab strains with respect to insecticide susceptibility (Lees et al, In Prep). F0 adults collected from larval breeding sites should only be used when lab strains are unavailable and should following the same insecticide resistance characterisation methods as lab strains (see Section 6. Deviations from standard protocol).
- For a pyrethroid-only test net panel: Use pyrethroid-susceptible mosquito strains (Lees et al, In Prep).
- For a pyrethroid + PBO test net panel: Use pyrethroid-susceptible and pyrethroid-resistant mosquito strains (Lees et al, In Prep).
- Where resources allow it and mosquitoes are available a second resistant and susceptible strain should be tested (See Section 5. Data priority list).
- For negative and positive control net panels: Test all mosquito strains being used on that experimental day against each control panel.

4.2. Collection and storage of test net samples (for bioefficacy testing[1])

- Whole nets and net pieces may need to be stored before and after testing and may be transported between study sites. When collecting and storing whole net and net samples always ensure they are kept separately to avoid cross-contamination of AIs. Store nets in a cool dry place at <5°C out of direct sunlight.
- Gloves and a lab coat should always be worn when handling the nets and should be changed between handling nets/net panels with different AIs to avoid cross-contamination.

[1] The number of sample pieces listed is for conducting the bioefficacy testing specified in this protocol. Additional samples may be required for chemical analysis.

- Hang sample net on net frame. Net frame should be cleaned between nets as specified by the labs cleaning protocols.
- Cut 4 pieces (30 x 30 cm) from each test net (2 from the roof panel, 2 from the sides panels). Scissors should be changed or cleaned between cutting net panels with different AIs. Recommended sampling positions can be found in Figure S1.
- Label net pieces with the sample position (i.e. 1 - 4) and net ID on paper labels secured to the corner of each piece.
- Wrap each piece individually in aluminium foil and refrigerate. If a refrigerator is not available store nets a cool dry place at <5°C.

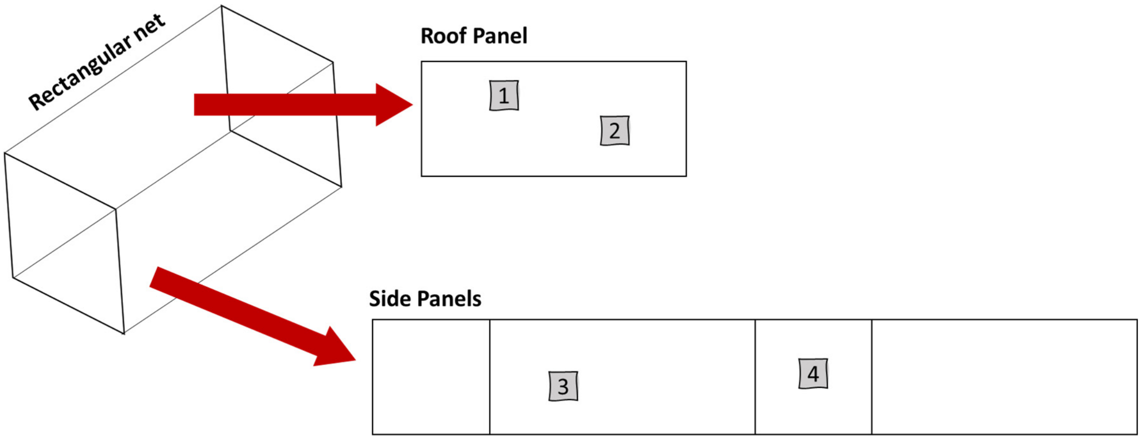

Figure S1. Recommended sampling position of net pieces from bednet. The lower 25 cm of the net should not be sampled as it is likely to have been exposed to abrasion from being tucked under a bed. Two samples should be taken from the net roof panel and two samples should be taken from the net side panels. Image adapted from (WHO, 2011).

4.3. Control net samples

- Gloves and a lab coat should always be worn when handling the nets and should be changed between handling different nets/net panels with different AIs to avoid cross-contamination.
- Control nets should be aired, but unwashed. Air new nets away from direct sunlight for a minimum of 7-days before testing.
- Only one piece of control netting is needed per assay. However, control pieces should not be used >5 times, so multiple pieces will be needed.
  - Hang net on net frame. Net frame should be cleaned between nets as specified by the labs cleaning protocols.
  - Cut 10 pieces (30 x 30 cm) from each control net.

- Label net pieces with the control net ID on paper labels and secure to the corner of each piece.
- Wrap each piece individually in aluminium foil and refrigerate. If a refrigerator is not available store nets a cool dry place at <5°C.

- On each experimental testing day, a negative control and two positive control nets (Table S1) should be tested alongside test nets.

Table S1. Specifications of control nets.

Net Type	Description
Negative control: Untreated net	Untreated netting of the same material as the test netting (e.g. polypropylene). Record the number of times the net piece has been used and do not use the same piece >5 times. If 24-hour mortality in the negative control on a particular testing day is >10% results should be discarded and testing repeated. If 24-hour control mortality for the day is <10% the test results should be corrected using Abbot's formula[2] (Abbott, 1925; WHO, 2013).
Positive control 1: New pyrethroid + PBO net panel	Brand new pyrethroid + PBO netting of the same brand as the test net. Air new nets away from direct sunlight for a minimum of 7-days before testing. Record the number of times the net has been used and do not use the same piece >5 times.
Positive control 2: New pyrethroid-only net panel	Brand new pyrethroid-only netting of the same material, treated using the same impregnation method, insecticide, and dose as the test netting. If such netting is not available, the closest non-PBO commercial equivalent should be used (e.g. if testing PermaNet 3.0, a new PermaNet 3.0 side panel could be used). Air new nets away from direct sunlight for a minimum of 7-days before testing. Record the number of times the net has been used and do not use the same piece >5 times.

[2] Abbott's formula: Adjusted mortality (%) = 100 x (X–Y) / (100–Y), where X is the percentage mortality with the test netting, and Y is the percentage mortality with the untreated control sample

4.4. Cone bioassay setup

- Gloves and a lab coat should always be worn when handling the nets and should be changed between handling different nets/net panels with different AIs to avoid cross-contamination.
- Clean testing area and equipment as specified by the labs cleaning protocols.
- Prepare test mosquitoes. The numbers of mosquitoes required for testing different types of pyrethroid + PBO net can be found in Table S3, Section 5. 'Data priority list'). Carefully transfer required mosquitoes to holding containers, 5 mosquitoes per container using an aspirator.
- Test mosquitoes and net samples should be acclimatised to the climatic conditions of the testing room for a minimum of one hour before testing. Remove any knocked-down mosquitoes from holding containers before testing.
- Prepare cone testing board(s).
    - Place 1st cone holder frame in stand.
    - Secure control and test nets to 1st cone holder frame with tape. Make sure nets do not overlap to avoid cross-contamination, that they are correctly labelled, and that the labels are visible.
    - Place the plastic cones over the nets and secure the cones in place by placing the 2nd cone holder frame over the top. The two cone holder frames can be secured together using binder clips or clamps.
    - Make sure that the board is stable and situated at a 45˚ angle.
    - Cover the opening of the plastic cones with a stopper (e.g. rubber plug or cotton wool).

4.5. Cone bioassay procedure

- Record the temperature and humidity during testing. Preferably continuously with a data logger, or alternatively manually at the start and end of exposure, and the end of the mosquito holding period.
- Exposed batches of 5 mosquitoes to netting pieces for 3 minutes for a total of 2 replicates per net piece:
    - Remove the stopper from the cone and transfer 5 mosquitoes from the holding container into the plastic cone using an aspirator. Take care not to touch the net with the aspirator end as this may result in contamination.
    - Cover the cone with the stopper to prevent mosquitoes from escaping.
    - Expose mosquitoes to the netting sample for 3 minutes.

  - Transfer mosquitoes from the cone back to their holding container with an aspirator. Take care not to touch the net with the aspirator end as this may result in contamination. Ensure containers are correctly labelled with the net sample ID (Net ID and position), test rep, mosquito species, and testing date.
  - Repeat until 2 replicates of 5 mosquitoes have been exposed to each net sample.
- Provide mosquitoes with a sugar meal (10% sucrose solution soaked onto a relevant substrate such as cotton wool).
- Record the number of mosquitoes in each holding container to give the total numbers exposed.
- After 1 hour post-exposure record the number of mosquitoes knockdown (Table S2).
- After 24 hours post-exposure record the number of dead mosquitoes (Table S2).
- At the end of testing, ensure mosquitoes are stored correctly (i.e. in individual tubes with silica gel) for future analysis. If mosquitoes are not required for future analysis, discard mosquitoes safely.

Table S2. The definitions used for classifying alive, knocked down or dead mosquitoes, adapted from (WHO, 2013)

Mosquito status	Definition
Alive	The mosquito is mobile or able to stand or fly in a coordinated manner
Knocked down	The mosquito is immobile or unable to stand or take off, at 1-hour following net exposure
Dead	The mosquito is immobile or unable to stand or take off, at 24-hours following net exposure

4.6. Measured outcomes

- The number of mosquitoes exposed, knocked down after 1-hour, and dead after 24-hours should be recorded for each net piece and replicate individually.
- For each panel type (i.e. pyrethroid-only panel or pyrethroid + PBO panels) sample and replicate data should be pooled and the 1-hour knockdown %[3] and 24-hour mortality %[4] calculated for each panel type.

[3] 1-hour knockdown (%) = (X/Y) x 100, where X is the total number of mosquitoes knocked down at 1-hour and Y in the total number of mosquitoes exposed to the test net.
[4] 24-hour mortality (%) = (X/Y) x 100, where X is the total number of mosquitoes dead at 24-hour and Y in the total number of mosquitoes exposed to the test net.

- Panel types should then be pooled to calculate the 1-hour knockdown %[5] and 24-hour mortality %[6] calculated for each net.

5. Data priority list

- All testing should be carried with the same resistant and susceptible strains over time. Where resources allow it and mosquitoes are available a second resistant and susceptible strains should be tested. However, it is more important to have a full data set with one strain, so resources should be prioritised to ensure this before considering testing with secondary strains.
- Ad hoc testing with secondary strains when available will provide useful data.
- The ideal methodological parameters (i.e. net samples, replicates, controls) can be found in Table S3. All methodological parameters and deviations from standard testing should be recorded at the time of testing.

Table S3. The number of mosquitoes required per test net and for daily controls for a pyrethroid + PBO net treated with PBO all over and for a pyrethroid + PBO net treated with PBO on the roof only. Numbers are based on testing 5 mosquitoes per replicate, with 2 replicates per net piece. For test nets 4 net pieces (2 from the roof, 2 from the sides) are used. For control nets 1 piece is used (3 control nets tested per day: untreated, new pyrethroid + PPO net, and new pyrethroid only net).

**Net with PBO on all panels**			
Strain	Test net	Daily control	Total
Resistant	40	30	70
Susceptible	40	30	70
Total	80	60	140
**Net with PBO only on roof panel**			
Strain	Test net	Daily control	Total
Resistant	20	30	50
Susceptible	40	30	70
Total	60	60	120

[5] 1-hour knockdown (%) = (X/Y) x 100, where X is the total number of mosquitoes knocked down at 1-hour and Y in the total number of mosquitoes exposed to the test net.
[6] 24-hour mortality (%) = (X/Y) x 100, where X is the total number of mosquitoes dead at 24-hour and Y in the total number of mosquitoes exposed to the test net.

6. Deviations from standard protocol

- All deviations from the standard protocol should be noted in the data collections sheets.
- When insecticide characterised lab strains are unavailable, wild larval collected mosquitoes could be used. Details on larval collected should be recorded, such as location of sampling sites (including co-ordinates), number of sampling sites, and type of sampling site (e.g. rainwater puddle, permanent water body). The wild larval collected population should be insecticide characterised using the same methods as those used to characterise lab strains (Lees et al, In Prep).

7. Supplementary data

- Additional information that should be recorded:
    - Time of testing
    - The light-dark rearing cycle of test mosquitoes (including times where possible)

8. Glossary of terms

AI	Active ingredient
I2I	Innovation to Impact
ITN	Insecticide-treated net
PBO	Piperonyl butoxide
PQ	Prequalification
SOP	Standard operating procedure
WHO	World Health Organization

9. References

Abbott, W.S. (1925) "A method of computing the effectiveness of an insecticide.," *Journal of Economic Entomology*, 18(2), pp. 265–267. Available at: http://www.ncbi.nlm.nih.gov/pubmed/3333059 (Accessed: January 4, 2017).

Lees, R. *et al.* (In Prep) "Strain Characterisation for Measuring Bioefficacy of ITNs Treated with Two Active Ingredients (Dual-AI ITNs) for Dura-bility Monitoring: Developing a Robust Protocol by Building Consensus," *Insects* [Preprint].

WHO (2011) *Guidelines for monitoring the durability of long-lasting insecticidal mosquito nets under operational conditions Control of Neglected Tropical Diseases WHO Pesticide Evaluation Scheme and Global Malaria Programme Vector Control Unit*.

WHO (2013) *Guidelines for laboratory and field-testing of long-lasting insecticidal nets, WHO/HTM/NTD/WHOPES/20131*. Geneva: World Health Organization.

WHO (2020) *List of WHO Prequalified Vector Control Products*. Available at: https://www.who.int/pq-vector-control/prequalified-lists/VCP_PQ-List_26August2020.pdf?ua=1 (Accessed: February 18, 2021).
