# Supplementary material for: Developing Consensus Standard Operating Procedures (SOPs) to Evaluate New Types of Insecticide-Treated Nets
Source: Insects. 2021 Dec 21;13(1):7. doi: 10.3390/insects13010007 (PMC8778287; doi:10.3390/insects13010007)
Supplement: Supplementary file 1 [file insects-13-00007-s001.zip › Additional File S3_PPF ITN Durability - SOP.pdf]

I2I-SOP-002: Methods for monitoring the durability of dual AI insecticide-treated nets containing a pyrethroid plus pyriproxyfen (PPF)

1. Purpose 1
2. Background 2
3. Materials & Equipment 2
4. Procedure 3
   4.1. Test mosquitoes 3
   4.2. Collection and storage of test net samples (for bioefficacy testing) 4
   4.3. Control nets samples 5
   4.4. Mosquito blood-feeding 6
   4.5. Cone bioassay setup 6
   4.6. Cone bioassay procedure 7
   4.7. Measuring sterility - Method for scoring oviposition using chambering. 8
   4.8. Measuring sterility - Method for scoring ovary development using ovary dissection 9
5. Data priority list 11
6. Deviations from standard protocol 12
7. Supplementary data 12
8. Glossary of terms 12
9. References 13

1. Purpose

This standard operating procedure (SOP) describes the methods to determine the bioefficacy of the pyrethroid and pyriproxyfen (PPF) components of insecticide-treated nets (ITNs) used under operational conditions. The process used to determine the methodology detailed in this SOP, and justifications for key methodological parameters can be found in 'I2I-MD-002: Durability monitoring method development: Dual AI insecticide-treated nets containing a pyrethroid plus pyriproxyfen

(PPF)I2I-MD-002: Durability monitoring method development: Dual AI ITNs containing pyriproxyfen'. This SOP details two methods to evaluate durability of pyrethroid + PPF nets, measuring mortality, and either (i) oviposition or (ii) dissection of ovaries following net exposure in a standard WHO cone bioassay. The same method should be used for all test nets throughout the durability trial.

2. Background

Pyrethroid + pyriproxyfen (PPF) nets are PQ listed (i.e. Royal Guard, **Error! Reference source not found.**) and being deployed in RCTs and pilot deployment schemes. The WHO cone test is a suitable method for exposing mosquitoes to pyrethroid + pyriproxyfen (PPF) nets for measuring the nets durability, but different endpoints are needed for each active ingredient. Knockdown and mortality can be used to assess the bio-efficacy of the pyrethroid but the most suitable endpoints for PPF, a juvenile hormone analogue that affects fertility and fecundity in mosquitoes, need to be defined. We are proposing two methods to evaluate durability of pyrethroid + PPF nets, measuring either (i) oviposition or (ii) dissection of ovaries following net exposure in a standard WHO cone bioassay. Monitoring the bioefficacy of the active ingredients (AI) in the nets is a vital part of establishing the durability of these nets under operational conditions.

3. Materials & Equipment

General

- Data collection sheets
- Lab coat
- Gloves
- Test pyrethroid + PPF nets
- Control untreated net
- Control new pyrethroid + PPF net
- Aspirator (manual/electronic), separate for each insecticide
- Mosquito strains
- Pen/permanent markers

Collection and storage of net samples

- Net frame
- Scissors
- Paper labels

- Aluminium foil

Cone bioassay

- Tape
- Mosquito holding containers (e.g. paper cups covered with untreated netting held by elastic bands)
- Cone holding frame (x 2), with holes to hold standard WHO plastic cones
- Cone holder frame stand, which holds frame at 45°
- WHO Plastic cones
- Binder clips or clamps
- Cotton wool or rubber stoppers
- Temperature and humidity data logger
- Timer
- 10% sucrose solution (e.g. sugar or honey and water)
- Cotton wool

Measuring sterility - Oviposition using chambering.

- Artificial egg laying chambers (e.g. falcon tubes containing damp water-soaked cotton wool covered with filter paper. Tube is covered with untreated netting secured in place with an elastic band)

Measuring sterility - Ovary development using ovary dissection

- Dissecting microscope
- Glass slides
- Dissection kit (e.g. dissection pins, forceps)
- Distilled water
- Plastic pipette/water dropper

4. Procedure

4.1. Test mosquitoes

- Use 3-to-5-day-old blood-fed female *Anopheles* mosquitoes. Mosquitoes should be blood-fed 3-9 hours prior to exposure. Mosquitoes should be well characterized lab strains with respect to insecticide susceptibility (Lees *et al*, In Prep). F_0 adults collected from larval breeding sites

should only be used when lab strains are unavailable and should following the same insecticide resistance characterisation methods (see Section 6. Deviations from standard protocol).

- A pyrethroid-susceptible strain should be tested to monitor the durability of the pyrethroid insecticide, and a pyrethroid-resistant strain should be used to monitor the durability of the PPF (Lees, *et al*, In Prep). Where resources allow it and mosquitoes are available a second resistant and susceptible strain should be tested (See Section 5. Data priority list).
- For negative and positive control net panels: Test all mosquito strains being used on that experimental day against each control panel.

4.2. Collection and storage of test net samples (for bioefficacy testing[1])

- Whole nets and net pieces may need to be stored before and after testing and may be transported between study sites. When collecting and storing whole net and net samples always ensure they are kept separately to avoid cross-contamination of AIs. Store nets in a cool dry place at <5°c out of direct sunlight.
- Gloves and a lab coat should always be worn when handling the nets and should be changed between handling different nets/net panels with different AIs to avoid cross-contamination.
- Hang sample net on net frame. Net frame should be cleaned between nets as specified by the labs cleaning protocols.
- Cut 4 pieces (30 x 30 cm) from each test net (2 from the roof panel, 2 from the sides panels). Scissors should be changed or cleaned between cutting net panels with different AIs. Recommended sampling positions can be found in Figure S1.
- Label net pieces with the sample position (i.e. 1 - 4) and net ID on paper labels secured to the corner of each piece.
- Wrap each piece individually in aluminium foil and refrigerate. If a refrigerator is not available store nets a cool dry place at <5°c.

[1] The number of sample pieces listed is for conducting the bioefficacy testing specified in this protocol. Additional samples may be required for chemical analysis.

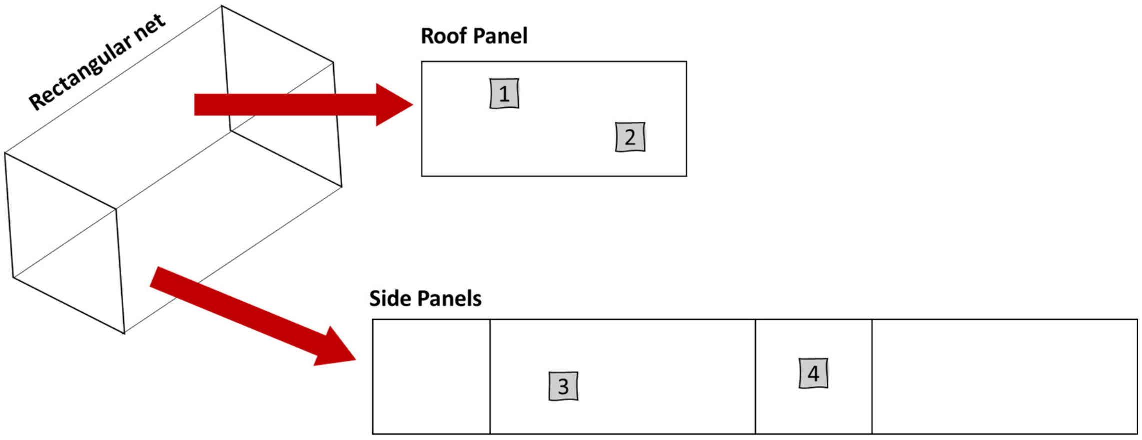

Figure S1. Recommended sampling position of net pieces from bednet. The lower 25 cm of the net should not be sampled as it is likely to have been exposed to abrasion from being tucked under a bed. Three samples should be taken from the net roof panel and three samples should be taken from the net side panels. Image adapted from (WHO, 2011).

4.3. Control nets samples

- Gloves and a lab coat should always be worn when handling the nets and should be changed between handling different nets/net panels with different AIs to avoid cross-contamination.
- Control nets should be aired, but unwashed. Air new nets away from direct sunlight for a minimum of 7-days before testing.
- Only one piece of control netting is needed per assay. However, control pieces should not be used >5 times, so multiple pieces will be needed.
    - Hang net on net frame. Net frame should be cleaned between nets as specified by the labs cleaning protocols.
    - Cut 10 pieces (30 x 30 cm) from each control net.
    - Label net pieces with the control net ID on paper labels and secure to the corner of each piece.
    - Wrap each piece individually in aluminium foil and refrigerate. If a refrigerator is not available store nets a cool dry place at <5°c.
- Only one piece of control netting is needed per assay. However, the methods for preparing control nets should follow the same procedure as test nets:
- On each experimental testing day, a negative control and a positive control net (Table S1) should be tested alongside test nets.

Table S1. Specifications of control nets.

Net Type	Description
Negative control: Untreated net	Untreated netting of the same material as the test netting (e.g. polypropylene). Record the number of times the net piece has been used and do not use the same piece >5 times. If 24-hour mortality in the negative control on a particular testing day is >10% results should be discarded and testing repeated. If 24-hour control mortality for the day is <10% the test results should be corrected using Abbot's formula[2] (Abbott, 1925; WHO, 2013).
Positive control: New pyrethroid + PPF net panel	Brand new pyrethroid + PFF netting of the same brand as the test net. Air new nets away from direct sunlight for a minimum of 7-days before testing. Record the number of times the net has been used and do not use the same piece >5 times.

4.4. Mosquito blood-feeding

- Blood-feed mosquitoes 3-9 hours before exposure. Mosquitoes should be blood fed using method of feeding standard for the test population (e.g. Hemotek membrane feeding system, arm feed, animal fed to repletion)
- Only visibly blood-fed[3] mosquitoes should be used for the assay.

4.5. Cone bioassay setup

- Gloves and a lab coat should always be worn when handling the nets and should be changed between handling different nets/net panels with different AIs to avoid cross-contamination.
- Clean testing area and equipment as specified by the labs cleaning protocols.
- Prepare test mosquitoes. The numbers of mosquitoes required can be found in Table S6, Section 5. Data priority list). Carefully transfer required mosquitoes to holding containers, 5 mosquitoes per container using an aspirator.

[2] Abbott's formula: Adjusted mortality (%) = 100 x (X–Y) / (100–Y), where X is the percentage mortality with the test netting, and Y is the percentage mortality with the untreated control sample.
[3] Visibly blood-fed mosquitoes: The mosquitoes' abdomen is engorged, and red. Filled with a bloodmeal and not a sugar meal.

- Test mosquitoes and net samples should be acclimatised to the climatic conditions of the testing room for a minimum of one hour before testing. Remove any knockdown mosquitoes from holding containers before testing.
- Prepare cone testing board(s).
    - Place 1st cone holder frame in stand.
    - Secure control and test nets to 1st cone holder frame with tape. Make sure nets do not overlap to avoid cross-contamination, that they are correctly labelled, and that the labels are visible.
    - Place the plastic cones over the nets and secure the cones in place by placing the 2nd cone holder frame over the top. The two cone holder frames can be secured together using binder clips or clamps.
    - Make sure that the board is stable and situated at a 45˚ angle.
    - Cover the opening of the plastic cones with a stopper (e.g. rubber plug or cotton wool).

4.6. Cone bioassay procedure

- Record the temperature and humidity during testing. Preferably continuously with a data logger, or alternatively manually at the start and end of exposure, and the end of the mosquito holding period.
- Exposed batches of 5 mosquitoes to netting pieces for 3 minutes for a total of 2 replicates per net piece:
    - Remove the stopper from the cone and transfer 5 mosquitoes from the holding container into the plastic cone using an aspirator. Take care not to touch the net with the aspirator end as this may result in contamination.
    - Cover the cone with the stopper to prevent mosquitoes from escaping.
    - Expose mosquitoes to the netting sample for 3 minutes.
    - Transfer mosquitoes from the cone back to their holding container with an aspirator. Take care not to touch the net with the aspirator end as this may result in contamination. Ensure containers are correctly labelled with the net sample ID (Net ID and position), test rep, mosquito species, and testing date.
    - Repeat until 2 replicates of 5 mosquitoes have been exposed to each net sample.
- Provide mosquitoes with a sugar meal (10% sucrose solution soaked onto a relevant substrate such as cotton wool). Ensure sugar meal is changed daily throughout assay, as this can impact mosquito health.
- Record the number of mosquitoes in each holding container to give the total numbers exposed.

- After 1 hour post-exposure record the number of mosquitoes knockdown (**Error! Reference source not found.**Table S2).
- After 24 hours post-exposure record the number of dead mosquitoes (Table S2).

Table S2. The definitions used for classifying alive, knocked down or dead mosquitoes, adapted from (WHO, 2013)

Mosquito status	Definition
Alive	The mosquito is mobile or able to stand or fly in a coordinated manner
Knocked down	The mosquito is immobile or unable to stand or take off, at 1-hour following net exposure
Dead	The mosquito is immobile or unable to stand or take off, at 24-hours following net exposure

4.7. Measuring sterility - Method for scoring oviposition using chambering.

- The sterilising effect of PPF can be measured in several ways. This SOP details two methods; (i) chambering to observe oviposition or (ii) ovary dissection to observe ovary development. Either method can be used, however the same method should be used for all nets throughout the durability trial.
- On Day 3 (three days post-exposure), record the number of dead mosquitoes in holding containers (Table S2).
- Set up artificial egg laying chambers using method used in your lab.
- Transfer alive mosquitoes individually into their own egg laying chamber using an aspirator.
- Ensure egg laying chambers are correctly labelled with a mosquito ID number, the net sample ID (Net ID and position), test rep, mosquito species, and testing date.
- Provide mosquitoes with a sugar meal (10% sucrose solution soaked onto a relevant substrate such as cotton wool).
- Place egg laying chambers in a dark area. Ensure sugar meal is changed daily throughout assay, as this can impact mosquito health.
- On Day 7 (four days post-chambering), record if each individual mosquito is alive or dead (Table S2). Record if each individual mosquito has laid eggs or not.
- At the end of testing, ensure mosquitoes are stored correctly (i.e. in individual tubes with silica gel) for future analysis. If mosquitoes are not required for future analysis, discard mosquitoes safely.

4.7.1. Measured outcomes

- The number of mosquitoes exposed, knocked down after 1-hour, and dead after 24-hours should be recorded for each net piece and replicate individually.
- Replicate data should then be pooled and the 1-hour knockdown %[4] and 24-hour mortality %[5] calculated for each individual net.
- On Day 3, number of mosquitoes dead, and number of alive mosquitoes chambered should be recorded for each test net.
- On Day 7, for each individual chambered mosquito status (alive or dead), and egg laying (laid eggs or did not lay eggs) should be recorded.
- Individual mosquito data should then be pooled to calculate oviposition and oviposition inhibition using the definition in Table S3.

Table S3. The definitions used for classifying oviposition and calculation of oviposition inhibition.

Mosquito status	Definition
Oviposition	$\frac{O}{T} \times 100$ Where *O* is the number of mosquitoes (living or dead) which laid eggs and *T* is the total number of surviving blood-fed mosquitoes placed into oviposition chambers.
Oviposition inhibition	$\frac{(Oc - Ot)}{Oc} \times 100$ Where Oc is the proportion of surviving blood-fed females from the control which laid eggs while Ot is the proportion of surviving blood-fed females from a given treatment which laid eggs.

4.8. Measuring sterility - Method for scoring ovary development using ovary dissection

- On Day 3 (three days post-exposure), record the number of dead mosquitoes in holding containers (Table S2).
- Dissect mosquito ovaries:
  - Mount mosquito onto glass slide on its back

[4] 1-hour knockdown (%) = (X/Y) x 100, where X is the total number of mosquitoes knocked down at 1-hour and Y in the total number of mosquitoes exposed to the test net.
[5] 24-hour mortality (%) = (X/Y) x 100, where X is the total number of mosquitoes dead at 24-hour and Y in the total number of mosquitoes exposed to the test net.

- Use a dissecting pin or forceps to hold the mosquito stationary by the thorax
- Add a drop of distilled water on to the last two segments of the mosquitoes abdomen.
- Use a dissection pin to gently pulling off the last two segments of the mosquito abdomen
- For better visualisation of the ovaries, use the needle to separate the ovaries from other internal material and wash off fat and other debris by rinsing the ovaries with distilled water.
- Leave the slide with dissected ovaries to air dry

- Two individuals should classify ovary development and they should be blinded to the net exposure treatment. Where disagreement on ovary classification occurs, a third individual should be consulted. If ovaries cannot be classified on the same day as dissection, photographs should be taken of the ovaries on the day and these should examined to classify ovaries.
- Record the developmental status of the eggs in each mosquitos' ovaries according to Christopher's stage of egg development (Figure S2).
- Record if the mosquito is fertile, Infertile, or inconclusive (Table S4)

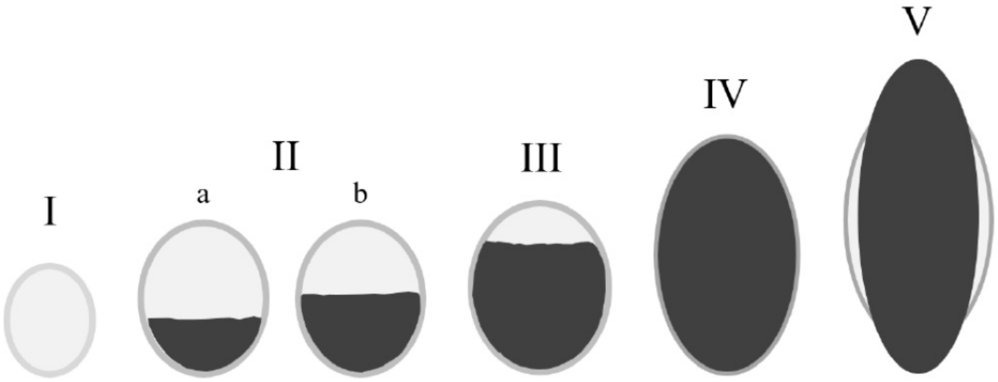

Figure 1. Christophers' Stages of Egg Development. Stage I: the primary follicle. Stage II: a) the follicle gains yolk protein, b) the follicle is approximately half comprised of yolk. Stage III: the follicle fills with yolk. Stage IV: the follicle elongates. Stage V: complete maturation of the egg, with floats.

Figure S2. Christopher's stages of egg development in female Anopheles mosquitoes, adapted from Christophers (1911).

Table S4. The definitions used for classifying egg development

Ovary status	Definition
Fertile	Female *Anopheles* eggs have fully developed to Christophers' stage V = normal elongated, boat/sausage-shaped eggs with lateral floats (Figure S2).

Infertile	Female *Anopheles* eggs have not fully developed and remain in Christophers' stages I –IV = less elongated, round shape, lacking floats (Figure S2).
Inconclusive	If both stage IV and stage V eggs are observed, record this as "inconclusive".

4.8.1. Measured outcomes

- The number of mosquitoes exposed, knocked down after 1-hour, and dead after 24-hours should be recorded for each net piece and replicate individually
- Replicate data should then be pooled and the 1-hour knockdown %[6] and 24-hour mortality %[7] calculated for each individual net.
- On Day 3, number of mosquitoes dead, and number of alive mosquitoes dissected should be recorded for each test net.
- For each dissected mosquito the developmental status of the eggs in each mosquitos' ovaries should be recorded, and this should be used to classify the mosquito as fertile, infertile or inconclusive.

5. Data priority list

- All testing should be carried with the same resistant and susceptible strains over time. Where resources allow it and mosquitoes are available a second resistant and susceptible strains should be tested. However, it is more important to have a full data set with one strain, so resources should be prioritised to ensure this before considering testing with secondary strains.
- Ad hoc testing with secondary strains when available will provide useful data.
- The ideal methodological parameters (i.e. net samples, replicates, controls) can be found in Table S5. When resources are reduced the number of mosquitoes required can be altered by changing the net samples required (Option B). All methodological parameters and deviations from standard testing should be recorded at the time of testing.

[6] 1-hour knockdown (%) = (X/Y) x 100, where X is the total number of mosquitoes knocked down at 1-hour and Y in the total number of mosquitoes exposed to the test net.
[7] 24-hour mortality (%) = (X/Y) x 100, where X is the total number of mosquitoes dead at 24-hour and Y in the total number of mosquitoes exposed to the test net.

Table S5. The number of mosquitoes required per test net and for daily controls. Examples are provided for a pyrethroid + PPF net treated with PPF all over. Green highlight is current ideal testing. Grey highlight shows suggested changes to reduce sample size.

		A	B
Roof panels samples		2	2
Side panel samples		2	1
Control samples (2 net types)		2	2
Replicate per panel		2	2
Mosquitoes per rep		5	5
Per test net	Resistant Strain	40	30
	Susceptible strain	40	30
Daily controls	Resistant Strain	20	20
	Susceptible strain	20	20
Total		120	100

6. Deviations from standard protocol

- All deviations from the standard protocol should be noted in the data collections sheets.
- When insecticide characterised lab strains are unavailable, wild larval collected mosquitoes could be used. Details on larval collected should be recorded, such as location of sampling sites (including co-ordinates), number of sampling sites, and type of sampling site (e.g. rainwater puddle, permanent water body). The wild larval collected population should be insecticide characterised using the same methods as those used to characterise lab strains (Lees, *et al*, In Prep).

7. Supplementary data

- Additional information that should be recorded:
  - Time of testing
  - The light-dark rearing cycle of test mosquitoes (including times where possible)

8. Glossary of terms

AI Active ingredient

I2I Innovation to Impact

ITN Insecticide-treated net

PPF	Pyriproxyfen
PQ	Prequalification
SOP	Standard operating procedure
WHO	World Health Organization

9. References

Abbott, W.S. (1925) “A method of computing the effectiveness of an insecticide.,” *Journal of Economic Entomology*, 18(2), pp. 265–267. Available at: http://www.ncbi.nlm.nih.gov/pubmed/3333059 (Accessed: January 4, 2017).

Lees, R. *et al* (In Prep) “Strain Characterisation for Monitoring Durability of Bioefficacy in ITNs Treated with Two Active Ingredients (Dual AI ITNs): Developing a Robust Protocol by Building Consensus,” *Insects* [Preprint].

WHO (2011) *Guidelines for monitoring the durability of long-lasting insecticidal mosquito nets under operational conditions Control of Neglected Tropical Diseases WHO Pesticide Evaluation Scheme and Global Malaria Programme Vector Control Unit*.

WHO (2013) *Guidelines for laboratory and field-testing of long-lasting insecticidal nets, WHO/HTM/NTD/WHOPES/20131*. Geneva: World Health Organization.
