# Supplementary material for: Developing Consensus Standard Operating Procedures (SOPs) to Evaluate New Types of Insecticide-Treated Nets
Source: Insects. 2021 Dec 21;13(1):7. doi: 10.3390/insects13010007 (PMC8778287; doi:10.3390/insects13010007)
Supplement: Supplementary file 1 [file insects-13-00007-s001.zip › Additional File S4_CFP ITN Durability - SOP.pdf]

I2I-SOP-003: Methods for monitoring the durability of dual AI insecticide-treated nets containing a pyrethroid plus Chlorfenapyr

1. Purpose ... 1
2. Background ... 2
3. Materials & Equipment ... 2
4. Procedure ... 3
4.1. Test mosquitoes ... 3
4.2. Collection and storage of test net samples (for bioefficacy testing) ... 3
4.3. Control net samples ... 4
4.4. Tunnel test setup ... 5
4.5. Tunnel test procedure ... 6
4.6. Measured outcomes ... 7
5. Data priority list ... 8
6. Deviations from standard protocol ... 9
7. Supplementary data ... 9
8. Glossary of terms ... 9
9. References ... 10

1. Purpose

This standard operating procedure (SOP) describes the methods to determine the bioefficacy of the pyrethroid and chlorfenapyr (CFP) components of insecticide-treated nets (ITNs) used under operational conditions. The process used to determine the methodology detailed in this SOP, and justifications for key methodological parameters can be found in 'I2I-MD-003: Durability monitoring method development: Dual AI insecticide-treated nets containing a pyrethroid plus Chlorfenapyr2I-MD-003: Durability monitoring method development: Dual AI ITNs containing Chlorfenapyr'.

2. Background

Pyrethroid + chlorfenapyr nets are PQ listed (i.e. Interceptor G2) and being deployed in randomised control trials (RCTs) and pilot deployment schemes. There is therefore an urgent need for a method to measure the bioefficacy of these nets, to collect baseline data and subsequently measure the durability of biological efficacy in nets collected from the field after fixed periods of use. Monitoring the bioefficacy of the active ingredients (AI) in the nets is a vital part of establishing the durability of these nets under operational conditions.

3. Materials & Equipment

General

- Data collection sheets
- Lab coat
- Gloves
- Test pyrethroid + CFP nets
- Control untreated net
- Aspirator (manual/electronic), separate for each insecticide
- Mosquito strains
- Pen/permanent markers

Collection and storage of net samples

- Net frame
- Scissors
- Paper labels
- Aluminium foil

Tunnel Test

- Mosquito holding/collection containers (e.g. paper cups covered with untreated netting held by elastic bands)
- 60 cm glass/plastic tunnels (25 cm x 25 cm square section), separate for each net type (i.e. untreated or dual-AI CFP net)
- Netted capture cages, 25 cm^2 (2 per tunnel)
- Net frame holder (1 per tunnel)
- Animal bait (specify what animal bait is being used)
- Temperature and humidity data logger

- Timer
- 10% sucrose solution (e.g. sugar or honey and water)

4. Procedure

4.1. Test mosquitoes

- Use 5-to-8-day-old nulliparous non-blood fed female *Anopheles* mosquitoes. Mosquitoes should be sugar starved for a minimum of 6 hours before exposure (the exact starvation period should be recorded). Mosquitoes should be well characterized lab strains with respect to insecticide susceptibility (Lees et al, In Prep). New IG1 & IG2 should be used to characterise strain prior to testing as these will not be used for daily controls.
- F_0 adults collected from larval breeding sites should only be used when lab strains are unavailable and should following the same insecticide resistance characterisation methods as lab strains (see Section 6. Deviations from standard protocol).
- A pyrethroid-susceptible strain should be tested to monitor the durability of the pyrethroid insecticide, and a pyrethroid-resistant strain should be used to monitor the durability of the CFP (Lees et al, In Prep). Where resources allow it and mosquitoes are available a second resistant and susceptible strain should be tested (See Section 5. Data priority list). As testing is conducted overnight, the different strains may need to be conducted on different days. Test all mosquito strains being used on that experimental day against each control panel.

4.2. Collection and storage of test net samples (for bioefficacy testing[1])

- Whole nets and net pieces may need to be stored before and after testing and may be transported between study sites. When collecting and storing whole net and net samples always ensure they are kept separately to avoid cross-contamination of AIs. Store nets in a cool dry place at <5°C out of direct sunlight.
- Gloves and a lab coat should always be worn when handling the nets and should be changed between handling nets/net panels with different AIs to avoid cross-contamination.

[1] The number of sample pieces listed is for conducting the bioefficacy testing specified in this protocol. Additional samples may be required for chemical analysis. However, it is advisable that the same samples be used for biological and chemical testing.

- Hang sample net on net frame. Net frame should be cleaned between nets as specified by the lab's cleaning protocols.
- Cut 2 pieces (20 x 20 cm) from each test net (1 from the roof panel, 1 from the sides panels). Scissors should be changed or cleaned between cutting net panels with different AIs. Recommended sampling positions can be found in Figure S1.
- Label net pieces with the sample position (i.e. 1 - 2) and net ID on paper labels secured to the corner of each piece.
- Wrap each piece individually in aluminium foil and refrigerate. If a refrigerator is not available store nets a cool dry place at <5°C.

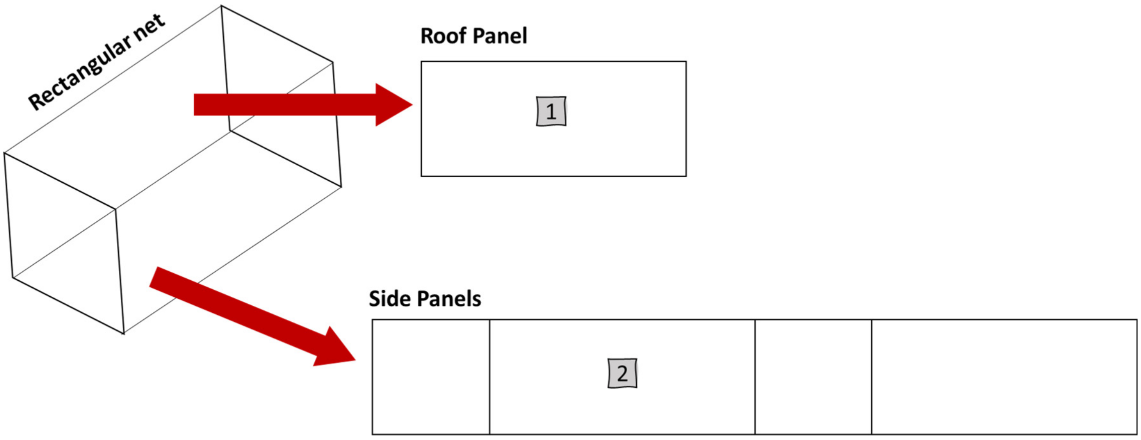

Figure S1. Recommended sampling position of net pieces from dual-AI CFP bednet, when the net is treated with the same AIs all over. The lower 25 cm of the net should not be sampled as it is likely to have been exposed to abrasion from being tucked under a bed. One sample should be taken from the net roof panel and one sample should be taken from the net side panels. Image adapted from (WHO, 2011).

4.3. Control net samples

- Gloves and a lab coat should always be worn when handling the nets and should be changed between handling different nets/net panels with different AIs to avoid cross-contamination.
- Control nets should be aired, but unwashed. Air new nets away from direct sunlight for a minimum of 7-days before testing.
- Two pieces of control netting are needed per assay. However, control pieces should not be used >5 times, so multiple pieces will be needed.
  - Hang net on net frame. Net frame should be cleaned between nets as specified by the labs cleaning protocols.

- Cut 10 pieces (20 x 20 cm) from each control net.
- Label net pieces with the control net ID on paper labels and secure to the corner of each piece.
- Wrap each piece individually in aluminium foil and refrigerate. If a refrigerator is not available store nets a cool dry place at <5°C.

- On each experimental testing day, two negative untreated control nets (Table S1) should be tested alongside test nets.

Table S1. Specifications of control nets.

Net Type	Description
Negative control: Untreated net	Untreated netting of the same material as the test netting (e.g. polypropylene). Record the number of times the net piece has been used and do not use the same piece >5 times. If mortality in the negative control on a particular testing day is >10% (24-hours) or >20% (72-hours) results should be discarded, and testing repeated. If control mortality for the day is <10% (24-hours) or <20% (72-hours) the test results should be corrected using Abbott's formula[2] (Abbott, 1925; WHO, 2013). If blood-feeding in the negative control on a particular testing day is <50% the results should be discarded and testing repeated.

4.4. Tunnel test setup

- Gloves and a lab coat should always be worn when handling the nets and should be changed between handling different nets/net panels with different AIs to avoid cross-contamination.
- Clean testing area and equipment as specified by the labs cleaning protocols.
- Prepare test mosquitoes. The numbers of mosquitoes required can be found in Table S4, Section 5. Data priority list). Carefully transfer required mosquitoes to holding containers, 50 mosquitoes per container using an aspirator.

[2] Abbott's formula: Adjusted mortality (%) = 100 x (X–Y) / (100–Y), where X is the percentage mortality with the test netting, and Y is the percentage mortality with the untreated control sample.

- Prepare test and control nets. Cut 9 holes into the 20 x 20 cm net piece (holes 1 cm in diameter; one hole is located at the centre of the square net sample, and the other eight are equidistant and located 5cm from the border). Secure the holed net onto the net holder.
- Test mosquitoes and net samples should be acclimatised to the climatic conditions of the testing room for a minimum of one hour before testing. Remove any knocked-down mosquitoes from holding containers before testing.
- Assemble the tunnel netted capture cages.
- Place the net sample in its holder into the tunnel, one third along its distance.
- Prepare and label 8 mosquito collection containers per tunnel, 4 for each compartment (compartment 1 & compartment 2[3]): labelled dead blood-fed, dead unfed, alive blood-fed, alive unfed.

4.5. Tunnel test procedure

- Record the temperature and humidity during testing. Preferably, continuously with a data logger or alternatively manually at the start and end of tunnel exposure, and the end of the mosquito holding period.
- Place the animal bait into compartment 2 (the shorter section of the tunnel). Ensure that the selected animal has not been used for testing for at least 2 weeks before this test and that it is placed in a position in the compartment where it will not injure itself.
- Secure the netted capture cages to either end of the tunnel. Ensure the netted cage on compartment 1 (the longer section of the tunnel) has an opening to allow the test mosquitoes to be added.
- Transfer 50 mosquitoes from the holding container into the netted cage on compartment 1 using an aspirator.
- Expose mosquitoes to the net in the tunnel for 12-15 hours. Record the start and end time of the exposure on the data collection sheet. Testing should be conducted in darkness during the 'night phase' of the mosquitoes circadian rhythm.
- At the end of the exposure period, determine the mosquitoes compartment location, mortality and blood-feeding status (Table S3). Transfer mosquitoes from the tunnel into their corresponding collection container using an aspirator (i.e. compartment 1 dead blood-fed). Return the animal to its enclosure and ensure it is provided with food and water.

[3] Compartment 1 = long section of tunnel into which mosquitoes are released; compartment 2 = short section where animal bait is housed.

- Ensure mosquito collection containers are correctly labelled with the net sample ID (Net ID and position), test rep, mosquito species, and testing date.
- Provide mosquitoes with a sugar meal (10% sucrose solution soaked onto a relevant substrate such as cotton wool).
- Record the number of mosquitoes in each holding container (i.e. compartment 1 or compartment 2, dead blood-fed, dead unfed, alive blood-fed, alive unfed).
- For remaining mosquitoes, record the number dead at 24, 48, and 72 hours from the start of exposure. Remove dead mosquitoes each day to avoid duplicate counting.
- At the end of testing, ensure mosquitoes are stored correctly (i.e. in individual tubes with silica gel) for future analysis. If mosquitoes are not required for future analysis, discard mosquitoes safely.

Table S2. The definitions used for classifying alive, dead, unfed and blood-fed mosquitoes, adapted from (WHO, 2013)

**Mosquito status**	**Definition**
Alive	The mosquito is mobile or able to stand or fly in a coordinated manner
Dead	The mosquito is immobile or unable to stand or take off in a coordinated manner following net exposure
Unfed	No blood-meal is visible by eye
Blood fed	A partial or complete blood-meal is visible by eye

4.6. Measured outcomes

- The number of mosquitoes in each compartment (1 & 2) on collection separated by dead blood-fed, dead unfed, alive blood-fed, alive unfed for each net/tunnel replicate.
- The number of mosquitoes dead at 24, 48, and 72hr, separated by their collection compartment (1 & 2) and blood-feeding status (unfed or blood-fed).

- Individual data should then be pooled to calculate blood-feeding %[4], blood-feeding inhibition[5], immediate mortality%[6], 24-hour mortality%[7] and 72-hour mortality %[8] for each net/tunnel replicate.
- If control mortality is >10% or blood-feeding is <50% for the day the test results should be discarded, and the test repeated.

5. Data priority list

- All testing should be carried with the same resistant and susceptible strains over time. Where resources allow it and mosquitoes are available a second resistant and susceptible strain should be tested. However, it is more important to have a full longitudinal data set with one strain, so resources should be prioritised to ensure this before considering testing with secondary strains.
- Ad hoc testing with secondary strains when available will provide useful data.
- The ideal methodological parameters (i.e. net samples, replicates, controls) can be found in **Error! Reference source not found.**. All methodological parameters and deviations from standard testing should be recorded at the time of testing.

---

[4] Blood-feeding (%) = (X/Y) x 100, where X is the total number of blood fed mosquitoes collected from the tunnel and Y in the total number of mosquitoes exposed to the test net in the tunnel.

[5] Blood feeding inhibition (%) = $\frac{(X-Y)}{X} \times 100$, where X is the blood feeding % in the untreated net tunnel and Y is the blood-feeding % in the test net tunnel.

[6] Immediate mortality (%) = (X/Y) x 100, where X is the total number of dead mosquitoes collected from the tunnel and Y in the total number of mosquitoes exposed to the test net in the tunnel.

[7] 24-hour mortality (%) = (X/Y) x 100, where X is the total number of mosquitoes dead within 24 hours and Y in the total number of mosquitoes exposed to the test net in the tunnel.

[8] 72-hour mortality (%) = (X/Y) x 100, where X is the total number of mosquitoes dead within 72 hours (including mosquitoes dead on collection and at 24- and 48-hours) and Y in the total number of mosquitoes exposed to the test net in the tunnel.

Table S3. The number of net samples, and mosquitoes required from each strain per test net and for daily controls.

Parameter	Amount
CFP roof panel sample	1
CFP side panel sample	1
Untreated control samples	2
Replicate per panel	1
Mosquitoes per rep	50
Mosquito per CFP test net	100
Mosquito per untreated control net (daily)	100
Total	200

6. Deviations from standard protocol

- All deviations from the standard protocol should be noted in the data collections sheets.
- When insecticide characterised lab strains are unavailable, wild larval collected mosquitoes could be used. Details on larval collected should be recorded, such as the location of sampling sites (including coordinates), the number of sampling sites, and the type of sampling site (e.g. rainwater puddle, permanent water body). The wild larval collected population should be insecticide characterised using the same methods as those used to characterise lab strains (Lees, et al, In Prep).

7. Supplementary data

- Additional information that should be recorded:
    - Time of testing
    - The light-dark rearing cycle of test mosquitoes (including times where possible)

8. Glossary of terms

AI	Active ingredient
CFP	Chlorfenapyr
I2I	Innovation to Impact
ITN	Insecticide-treated net

PBO	Piperonyl butoxide
PQ	Prequalification
RCT	Randomised control trial
SOP	Standard operating procedure
WHO	World Health Organization

9. References

Abbott, W.S. (1925) "A method of computing the effectiveness of an insecticide.," *Journal of Economic Entomology*, 18(2), pp. 265–267. Available at: http://www.ncbi.nlm.nih.gov/pubmed/3333059 (Accessed: January 4, 2017).

Lees, R. *et al* (In Prep) "Strain Characterisation for Monitoring Durability of Bioefficacy in ITNs Treated with Two Active Ingredients (Dual AI ITNs): Developing a Robust Protocol by Building Consensus," *Insects* [Preprint].

WHO (2011) *Guidelines for monitoring the durability of long-lasting insecticidal mosquito nets under operational conditions Control of Neglected Tropical Diseases WHO Pesticide Evaluation Scheme and Global Malaria Programme Vector Control Unit*.

WHO (2013) *Guidelines for laboratory and field-testing of long-lasting insecticidal nets, WHO/HTM/NTD/WHOPES/20131*. Geneva: World Health Organization.
